# A Density Peak-Based Method to Detect Copy Number Variations From Next-Generation Sequencing Data

**DOI:** 10.3389/fgene.2020.632311

**Published:** 2021-01-13

**Authors:** Kun Xie, Ye Tian, Xiguo Yuan

**Affiliations:** ^1^The School of Computer Science and Technology, Xidian University, Xi’an, China; ^2^Xi’an Key Laboratory of Computational Bioinformatics, The School of Computer Science and Technology, Xidian University, Xi’an, China

**Keywords:** copy number variations, next-generation sequencing data, density peak, null distribution, read depth

## Abstract

Copy number variation (CNV) is a common type of structural variations in human genome and confers biological meanings to human complex diseases. Detection of CNVs is an important step for a systematic analysis of CNVs in medical research of complex diseases. The recent development of next-generation sequencing (NGS) platforms provides unprecedented opportunities for the detection of CNVs at a base-level resolution. However, due to the intrinsic characteristics behind NGS data, accurate detection of CNVs is still a challenging task. In this article, we propose a new density peak-based method, called dpCNV, for the detection of CNVs from NGS data. The algorithm of dpCNV is designed based on density peak clustering algorithm. It extracts two features, i.e., local density and minimum distance, from sequencing read depth (RD) profile and generates a two-dimensional data. Based on the generated data, a two-dimensional null distribution is constructed to test the significance of each genome bin and then the significant genome bins are declared as CNVs. We test the performance of the dpCNV method on a number of simulated datasets and make comparison with several existing methods. The experimental results demonstrate that our proposed method outperforms others in terms of sensitivity and F1-score. We further apply it to a set of real sequencing samples and the results demonstrate the validity of dpCNV. Therefore, we expect that dpCNV can be used as a supplementary to existing methods and may become a routine tool in the field of genome mutation analysis.

## Introduction

Copy number variation (CNV) is an important category of DNA structural variations, including amplifications or losses of DNA fragments with a length of more than 1 kilo base-pairs (bp) (Freeman et al., 2006; Yuan et al., 2012b). The mutation rate of CNV loci is much higher than that of single nucleotide polymorphisms (SNP) across the whole genome. CNV is one of the important pathogenic factors affecting human complex diseases (Shlien and Malkin, 2009; Fridley et al., 2012; Xi et al., 2020a,b). Therefore, it is necessary and meaningful to analyze CNVs when studying and treating complex diseases especially human cancers. Generally, the mechanisms for the formation of CNVs can be classified into two categories: DNA recombination and DNA error replication (Martin et al., 2019). In each category of the mechanisms, CNVs are usually presented in either amplification or deletion states. The major step of CNV analysis in samples obtained from human cancers is to identify which genome regions are CNVs and determine the corresponding states (i.e., either amplification or deletion). Therefore, it is required to develop statistically computational methods to analyze the data generated by different sequencing technologies.

There are three primary types of technologies that can produce data sets for the detection of CNVs: array comparative genomic hybridization (aCGH), SNP array, and next-generation sequencing (NGS) technologies. Currently, various computational methods have already been developed for analyzing each type of the data sets. For example, aiming at aCGH data, classic methods include fastRPCA (Nowak et al., 2011), PLA (Zhou et al., 2014), WaveDec (Cai et al., 2018), and graCNV (Auer et al., 2007). Meanwhile, aiming at SNP array data, famous methods include GISTIC (Beroukhim et al., 2007), STAC (Diskin et al., 2006), SAIC (Yuan et al., 2012b), and AISAIC (Zhang et al., 2014). In comparison with these two types of data, NGS data is at the highest resolution and is used widely for the detection of CNVs in recent years. Due to the inherent characteristics behind NGS data, the CNV detection methods using NGS data can be classified into four categories (Zhao et al., 2013): pair-end mapping, split-read, *de no* assembly, and read depth (RD) based approaches. The intention of the pair-end mapping-based approach is that it determines CNVs according to the difference of the length between the two ends of paired reads mapped to the reference and the insert fragment, while the split-read based approach determines CNVs by splitting the sequence and observing the distance of the split reads mapped to the reference sequence. *De no* assembly approach is usually used to find out novel inserted sequences (Yuan et al., 2019b). These three categories of approaches are appropriate for the detection of CNVs with a limited size, since the pair-end mapping and split-read based approaches are subject to the length of inserted fragments and the *de no* assembly method is subject to the cost of computation time. Nevertheless, CNVs are usually ranging at a large scope of interval in size, and can be up to more than tens of M base-pairs. Relative to the above three categories, the RD based approach is more versatile in detecting CNVs with any sizes. The major principle of this approach is to determine CNVs according to the variance of RDs across the genome to be analyzed.

The RD based approach is generally implemented through the following four steps (Duan et al., 2014; Yuan et al., 2019a): (1) mapping sequencing reads to a reference genome and extracting a read count profile, (2) dividing the genome into non-overlapping bins and calculating a RD value for each bin based on the read count profile, (3) making normalization and correction to the RD values, and (4) analyzing the corrected RD values to declare CNVs. The theoretical assumption underlying the RD based approach is that the RD value of one bin or one region is roughly related to its corresponding copy number, i.e., the larger the RD value, the larger the copy number, and vice versa. Therefore, the key point here is how to design an appropriate scheme to reasonably analyze the RD values. The currently popular methods for detecting CNVs using RD values include but are not limited to: RDXplorer (Yoon et al., 2009), CNVnator (Abyzov et al., 2011), GROM-RD (Smith et al., 2015), XCAVATOR (Magi et al., 2017), Control-FREEC (Boeva et al., 2012), CNVkit (Talevich et al., 2016), CNAseg (Ivakhno et al., 2010), CopywriteR (Kuilman et al., 2015), SeqCNV (Chen et al., 2017), CloneCNA (Yu et al., 2016), iCopyDAV (Dharanipragada et al., 2018), DeAnnCNV (Zhang et al., 2015), CNV_IFTV (Yuan et al., 2019c), CONDEL (Yuan et al., 2020), and CNV-LOF (Yuan et al., 2019a). Each of these methods has its own characteristics and advantages. For example, Control-FREEC makes the best use of GC-content to normalize the read count profile so as to find out CNV regions, and iCopyDAV chooses an appropriate bin size and uses thresholds for RD values to declare CNVs. Although much effectiveness has been achieved by these methods, some factors such as low-level tumor purity (i.e., the fraction of tumor cells in the sequencing sample), limited coverage depth and GC-content bias still pose a big challenge to the detection of CNVs with small amplitudes. Therefore, it would be necessary and meaningful to seek for new methods that can grasp the essential characteristics of sequencing data associated with CNVs.

Given the above, we summarize several aspects that should be considered to improve the detection of CNVs. In the first place, it is necessary to make a smooth or segmentation to the observed RD profile, so that adjacent bins with similar amplitudes can be merged into the same region and the bins showing a local mutation state cannot be masked. In the second place, it is meaningful to extract effective features from sequencing data that can make an accurate distinguishing between mutated and normal genome regions. In the last place, it is necessary to design a reasonable model for displaying the extracted features and perform a suitable analysis of the features to determine CNVs.

With a careful consideration of the problems described above, in this article, we propose a new method, called dpCNV, for the detection of CNVs from NGS data. The motivation and underlying idea of dpCNV could be demonstrated as below. It considers the inherent correlations among adjacent positions on the genome, and thus analyzes CNVs based on the unit of genome segments rather than individual bins. These segments can be produced by performing a segmentation process on the RD profile. It carefully takes into account that CNV regions usually accounts for a small fraction of the whole genome and many CNVs just display a “local” outlier state, and thus extracts two related features (i.e., local density and minimum distance) from the RD profile based on the density peak algorithm (Rodriguez and Laio, 2014). Finally, dpCNV analyzes the two feature values for each segment through multivariate Gaussian distribution and calculates the corresponding *p*-value to declare whether it is a CNV. We perform a large number of simulation experiments to test the dpCNV method and make comparisons with several existing methods. The experimental results demonstrate the merit of the proposed method. Moreover, we apply it to analyze a set of real sequencing samples and prove its validity.

The remainder of this article is organized as follows. Section “Materials and Methods” demonstrates the workflow of dpCNV and the related principles. In section “Results,” simulation studies are designed to evaluate the performance of the proposed method and its peer methods, as well as validations by applying it to a set of real sequencing samples. Section “Conclusion” discusses the proposed method and summarizes an outline of future work.

## Materials and Methods

### Workflow of dpCNV

**FIGURE 1:**
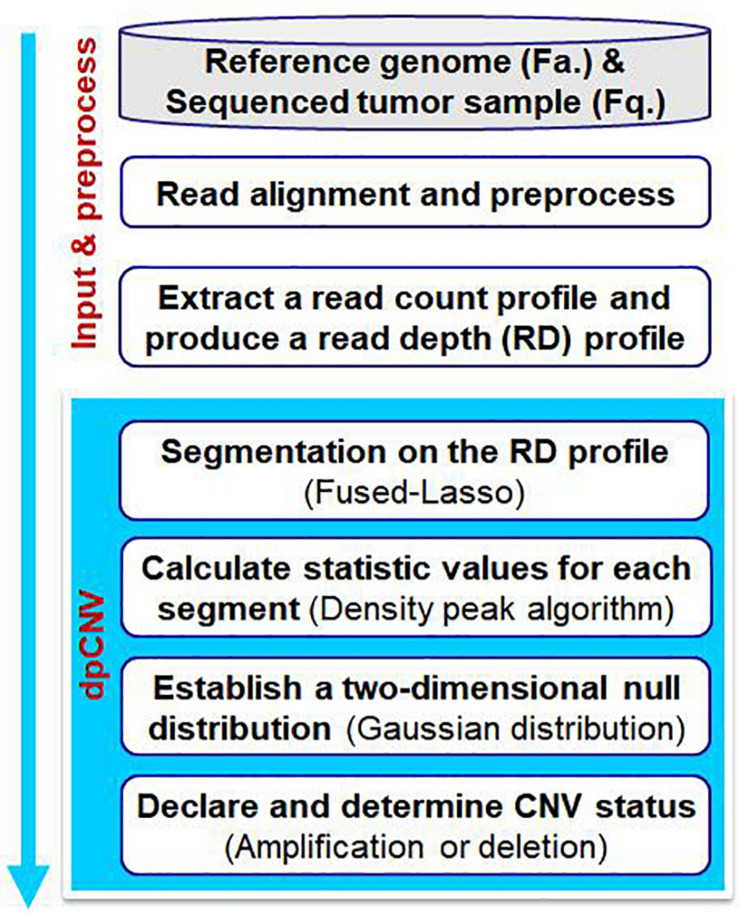
Workflow of the dpCNV method to detect copy number variations from tumor samples with next-generation sequencing data.

The workflow of the dpCNV method is demonstrated in Figure 1. The dpCNV method works by starting from an input of a sequenced tumor sample and a reference genome. The sequenced tumor sample is aligned to the reference genome by using the commonly used alignment tool BWA (Li and Durbin, 2009), and then a read count profile is extracted from the alignment result by using SAMtools (Li et al., 2009). With the read count profile, a RD profile is produced with a pre-defined bin size, such as 1000 base pairs (bp), which is moderate in the detection of CNVs (Yuan et al., 2020).

Based on the RD profile, the dpCNV method performs CNV analysis via the following four steps. (I) It implements a segmentation process on the RD profile to generate small genome segments, each of which usually include a set of adjacent and correlated bins. Here, the segmentation is carried out by using the Fused-Lasso algorithm (Tibshirani and Wang, 2008). (II) It extracts two features as the statistic and calculates the corresponding values via density peak algorithm. (III) It establishes a two-dimensional null distribution via multivariate Gaussian distribution and tests significance for each segment. (IV) It declares CNVs via a threshold of significance level and determines CNV statuses (i.e., amplification or deletion) via a RD cutoff.

### Segmentation on the RD Profile

With the RD profile, a GC-content bias correction process is carried out through a similar approach with the works (Abyzov et al., 2011; Yuan et al., 2019a), and then a segmentation process is implemented on the corrected RD profile. The purpose of the segmentation is to divide the whole RD profile into a set of small segments, each of which is composed by adjacent bins, and is to provide a segment-based unit for the detection of CNVs rather than a bin-based unit. Theoretically, the segment-based unit can help to increase the independence of elements in significance testing, so that a reasonable evaluation of *p*-values can be expected to be achieved (Yuan et al., 2012b). Nevertheless, the bin-based unit may result in a conservativeness of *p*-value evaluation since adjacent bins are usually correlated (Yuan et al., 2019c).

There are various existing approaches that can carry out segmentation on the RD profile. Here we choose the Fused-Lasso algorithm for this task (Tibshirani and Wang, 2008). In comparison with other segmentation algorithms, the Fused-Lasso algorithm performs better in smoothing adjacent bins with highly similar RD values while remaining local fluctuations among the resulted segments (Tibshirani and Wang, 2008). For convenience, the resulted segments are denoted by:

(1) $$S=\left\{s_{1},s_{2},s_{3},\ldots ,s_{n}\right\}$$

where *n* denotes the total number of segments that have been achieved. The following steps of analyzing CNVs are based on the set of *S*.

### Calculation of Statistic Values for Each Segment

With the segment-based RD profile *S*, we adopt the density-based peak algorithm to extract two features as the statistic for each segment: local density (ρ) and minimum distance (δ), and to calculate their corresponding values. With the consideration of that regions with changed copy numbers are inherently different from those of normal copy numbers and only account for a small part of the whole genome, we transfer the problem of detecting CNVs to the issue of identifying outliers from the set of segments with features of ρ and δ. Accordingly, each segment can be regarded as an object or a point in the two dimensional space of ρ and δ. In the following text, we make a detailed description to these two features and the calculation approach.

Before describing the two features ρ and δ, we introduce the Euclidean distance between any two objects (segments) *s*_*i*_ and *s*_*j*_. Given the total number of segments of *n*, an Euclidean distance matrix *M*_*n×n*_ can be obtained, where each element (*d*_*ij*_) can be calculated by the Euclidean distance formula:

(2) $$d_{i\,j}=\sqrt{{\left(\rho_{i}-\rho_{j}\right)}^{2}+{\left(\delta_{i}-\delta_{j}\right)}^{2}}$$

where ρ_*i*_ and δ_*i*_ represent the feature values of object *s*_*i*_, and the same to ρ_*j*_ and δ_*j*_. With the Euclidean distance matrix *M*_*n×n*_, an adjustable distance threshold γ is introduced according to the theorem of the density peak algorithm (Rodriguez and Laio, 2014). This threshold can be explained as a radius of each object *s*_*i*_ and is used to calculate how many objects are adjacent to the object *s*_*i*_ within the distance of γ. Then, the concept of local density ρ for each object is produced.

#### Definition 1

The local density ρ_*i*_ of the object *s*_*i*_ is defined as the number of objects adjacent to the object *s*_*i*_ with the radius γ, and can be calculated by using Eq. 3:

(3) $$\rho_{i}=\sum_{j\neq i}^{n}\chi \,\left(d_{i\,j}-\gamma \right)$$

where χ(*x*) = 1 if *x* < 0, and otherwise, χ(*x*) = 0.

#### Definition 2

The minimum distance δ_*i*_ of the object *s*_*i*_ is defined as the minimum value among the distances between the object *s*_*i*_ and those objects with higher density than *s*_*i*_, and can be expressed as Eq. 4:

(4) $$\delta_{i}=\min_{j:\rho_{i}<\rho_{j}}\left(d_{i\,j}\right).$$

For the object *s*_*i*_ with the highest density, the value δ_*i*_ is defined as the maximum distance between the object and the rest of objects in the set *S*, and can be expressed as Eq. 5:

(5) δi=maxj⁡(di⁢j) if ρi≥ρjj≠ij.

**FIGURE 2:**
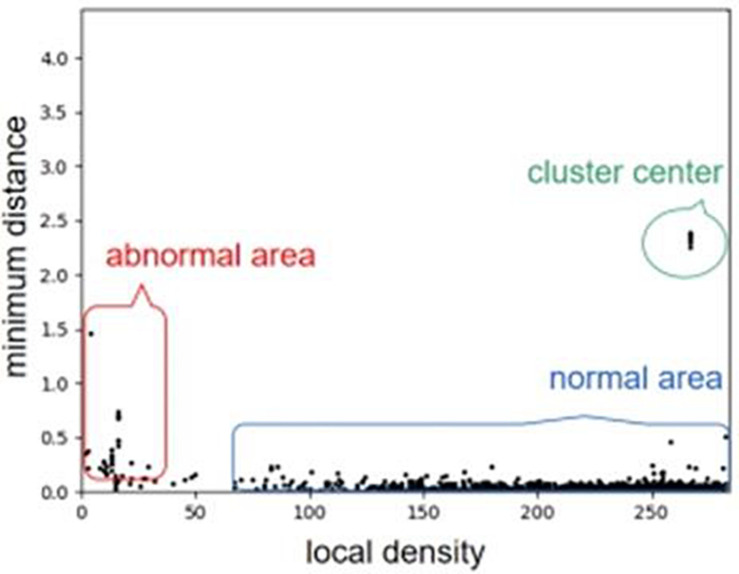
An example of describing the distribution of a set of objects with respect to the values of the two features. We can note that the objects at the abnormal area (outliers) are near to the left and bottom side of the distribution.

For a clear understanding of local density and minimum distance, we use an example to describe the distribution of a set of objects with respect to the values of the two features, as shown in Figure 2. For the example, we can see that the objects at the abnormal area (outliers) are near to the left and bottom side of the distribution. From the basic idea of density peak algorithm, outliers usually have a larger minimum distance and a smaller local density than those of other objects. Here, the abnormal area denotes the place of outlier objects, and normal area denotes the cluster of most objects. More details about the density peak algorithm is referred to Rodriguez and Laio (2014).

### Establish of a Two-Dimensional Null Distribution

With the statistic values in a two-dimensional space [i.e., local density (ρ) and minimum distance (δ)], the task now is how to design an appropriate model to test the significance of them. Since the values of the two features are usually at different scopes, it is not appropriate to combine them as a single feature value for the analysis. Therefore, it would be reasonable to design a model that can analyze the statistic values in a two-dimensional space. To mirror this, we establish a multivariate (i.e., two-dimension) Gaussian distribution as the null distribution based on the observed statistic values, and then evaluate a *p*-value for each of them. The multivariate Gaussian distribution is expressed as Eq. 6:

(6) $$p\,\left(x;\mu ,\Sigma \right)=\frac{1}{\left(2\,\pi \right)\,{\left|\Sigma \right|}^{\frac{1}{2}}}\,\exp\left(-\frac{1}{2}\,{\left(x-\mu \right)}^{T}\,\Sigma^{-1}\,\left(x-\mu \right)\right)$$

where μ is a two-dimensional vector, representing the mean values of local density and minimum distance, i.e., $\mu =\left[\bar{\rho ,\delta }\right]$, and Ʃ represents the covariance matrix of the two features.

**FIGURE 3:**
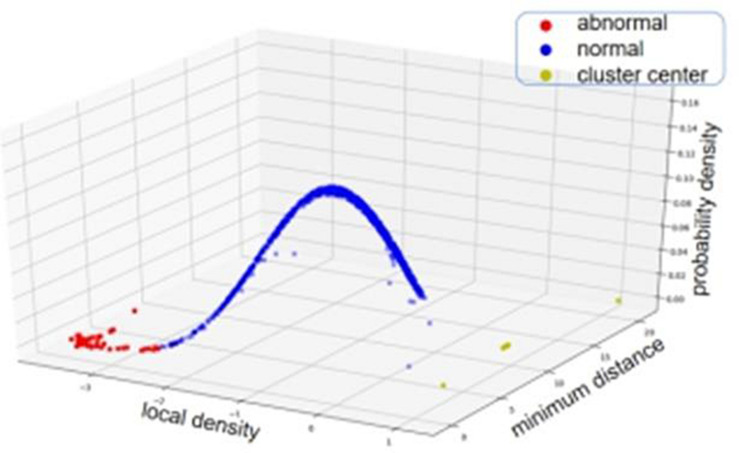
An example of showing the two-dimensional Gaussian distribution of the statistic values (i.e., local density and minimum distance) based on simulation data. The blue points represent the segments with normal copy numbers while the red points represent the segments with abnormal copy numbers.

The reason about why to choose a multivariate Gaussian distribution as the null distribution can be explained as below. Assuming that there are no CNVs in the segment-based RD profile *S*, and then the mean RD value should be around the sequencing coverage depth of the whole genome and the variance is primarily contributed by random artifacts such as sequencing and mapping errors. From this viewpoint, the RD values can be approximately modeled by a Gaussian distribution (Yuan et al., 2020). Theoretically, with a Gaussian distributed object, the deduced local density (ρ) and minimum distance (δ) would also follow Gaussian distribution, respectively. Therefore, the joint of the two features can be approximately modeled by a two-dimensional Gaussian distribution. For a clear understanding of this, we depict an example using a simulated dataset to show the distribution of the statistic values (ρ, δ) in Figure 3.

### Declaration and Determination of CNV Statuses

Based on the two-dimensional null distribution above, the *p*-value (*p*_*i*_) for each object (segment) *s*_*i*_ can be calculated. We define a commonly used significance level α as the cutoff for declaring CNVs, i.e., if *p*_*i*_ is less than α, then the object *s*_*i*_ will be declared as a CNV status; otherwise, it is regarded as a normal status. According to our experience and a large number of simulation experiments, we find that the value of α is appropriate to be assigned with 0.005.

With the abnormal objects, we further deduce their types (i.e., amplification or deletion) of CNV according to their RD values. Here, we use the average RD value of the objects in the cluster center (shown in Figure 3) as the baseline (*r*_*b*_) of normal copy number. This is consistent with that the objects in the cluster center are regarded as normal objects according to the density peak algorithm. Subsequently, for each abnormal object, if its RD value is larger than *r*_*b*_, then it is regarded as an amplification event, otherwise, it is regarded as a deletion event.

## Results

The dpCNV software is implemented in Python language, and the code is publicly available at https://github.com/BDanalysis/dpCNV/. In order to demonstrate the performance and usefulness of our proposed method, we first conduct a number of simulation experiments and make comparisons with several existing methods in terms of precision, sensitivity and F1-score (the harmonic mean of sensitivity and precision). Then, we apply the proposed method to a set of real sequencing samples, which have been obtained from the European Genome-phenome Archive (EGA) databases.^1^ To assure a fair comparison between dpCNV and other methods, we use the default parameter values in the implementation of the compared methods.

## Simulation Studies

Simulation studies are usually regarded as an appropriate and feasible way to assess the performance of existing and newly developed methods (Yuan et al., 2012a, 2017, 2018). This is because that the ground truth CNVs embedded in the simulated data sets could be used for an exact calculation of sensitivity and precision for the methods. Currently, there are many methods for simulating NGS data have been proposed. Here, we use one of our previously developed simulation methods, IntSIM (Yuan et al., 2017), for the simulation of NGS data with ground truth CNVs. Two primarily factors (i.e., tumor purity and depth of coverage) have been considered in the simulation process. Specifically, six scenarios have been simulated by setting different values of tumor purity (0.2, 0.3, and 0.4) and coverage depth (4× and 6×), and in each scenario 50 replicated samples have been produced.

**FIGURE 4:**
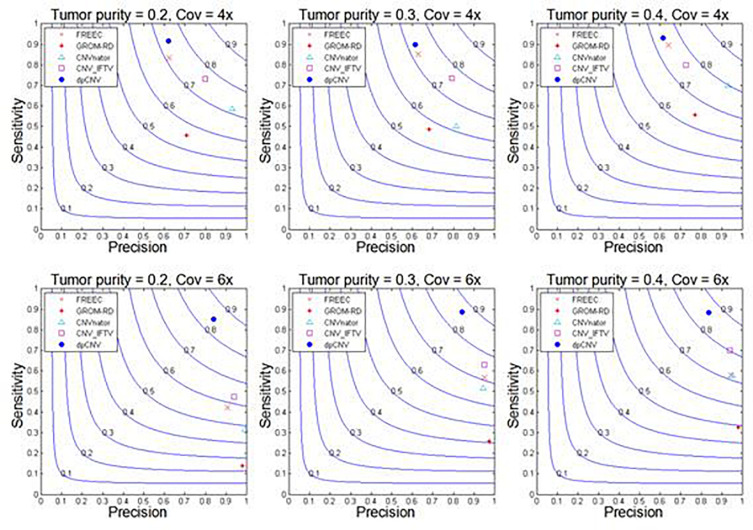
Performance comparisons between our proposed method and the four peer methods in terms of sensitivity, precision, and F1-score (colored curves) on simulation data.

With these simulated data sets, the dpCNV method and four peer methods (including FREEC, GROM-RD, CNVnator, and CNV_IFTV) are performed. Their results and comparisons are depicted in Figure 4. Here, the precision is calculated as the ratio of the number of correctly detected CNVs to the number of all declared CNVs, while the sensitivity is calculated by the ratio of the number of correctly detected CNVs to the total number of ground truth CNVs. From the Figure 4, one could observe that the performances of most methods are improving along with the increasing of tumor purity and coverage depth. Comparatively, the dpCNV method is superior in terms of the trade-off (F1-score) between precision and sensitivity in each of the simulation scenarios. With respect to sensitivity, dpCNV ranks first in all the simulation scenarios, followed by FREEC or CNV_IFTV. With respect to precision, GROM-RD and CNVnator display larger values than other methods.

The fact that dpCNV is superior to other methods under this study is due to the following reasons. Firstly, the relationship between adjacent bins has been taken into account by performing a segmentation process. In this process, most noised data points can be smoothed, and some local variations can be remained. In addition, two meaningful features (i.e., local density and minimum distance) are extracted from the segmented data based on a density peak algorithm. Secondly, a two-dimensional null distribution has been established for testing the significance of each genome segment. This can help to relieve the conservativeness of *p*-value assessment and provide a meaningful null hypothesis testing.

### Real Data Applications

**FIGURE 5:**
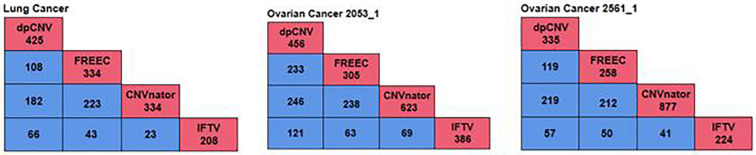
The overlapping results of four methods on the three samples. The red boxes represent the total number of CNVs detected by each method, while the blue boxes denote the number of overlapping CNVs detected by any two methods.

To further validate the performance of dpCNV, we apply it to three whole-genome sequencing data (EGAD00001000144_LC, EGAR00001004802_2053_1, and EGAR00001004836_2561_1) obtained from the EGA project. These samples include a lung cancer sample and two ovarian cancer samples. Besides, we also perform three peer methods (FREEC, CNVnator, CNV_IFTV) on these samples for comparisons. Since real sequencing data usually have no ground truth CNVs, it is difficult for us to exactly calculate the sensitivity and precision for the methods. Nevertheless, we analyze the overlapping results among the compared methods to observe the consistence between their results, as shown in Figure 5. We can note that CNVnator gets the largest number of overlaps with other methods, followed by dpCNV and FREEC. However, the total number of detected CNVs detected by CNVnator is also the largest. This means that it is not appropriate to determine which method is superior just according to the number of overlapped CNVs. Nevertheless, we adopt the overlapping density score (ODS) proposed in our previous work (Yuan et al., 2020) to evaluate the methods. The ODS is calculated by using Eq. 7. The comparative result is shown in Table 1, from which we can notice that dpCNV achieves the highest ODS in the analysis of two ovarian tumor samples and FREEC gets the highest ODS in the analysis of the lung tumor sample:

(7) $$\text{ODS}=m_{c\,n\,v}\cdot m_{c\,n\,v}^{\prime }$$

**TABLE 1:** Comparison of ODS between dpCNV and three peer methods on real samples.

Sample	dpCNV	FREEC	CNV_IFTV	CNVnator
EGAD00001000144_LC	99.4	**114.02**	47.96	19.06
EGAR00001004802_2053_1	**155.25**	152.89	35.75	44.2
EGAR00001004836_2561_1	**263.16**	192.79	57.04	114.7
Average	**172.6**	153.23	46.92	59.32

*Bold value denotes the largest values in each line.*

where *m*_*cnv*_ denotes the total overlapped CNVs divided by the number of compared methods and $m_{c\,n\,v}^{\prime }$ denotes the total overlapped CNV divided by the number of CNVs detected by itself.

**FIGURE 6:**
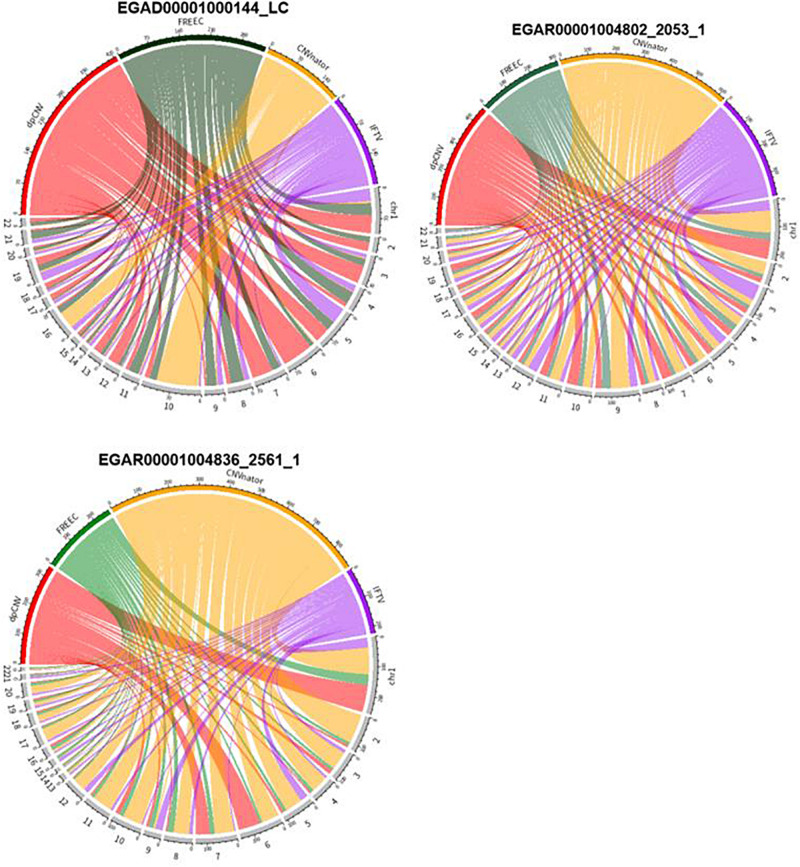
The circus diagram on three real samples. The upper part consisting of four arcs indicates the four methods, while the lower part consisting of 22 arcs denotes 22 autosomes. The length of each arc in upper part represents the total number of detected CNVs.

An overview of the numbers of CNVs detected by the four methods are shown in Figure 6, where we could clearly take an overview of distribution on 22 autosomes of results called by dpCNV, FREEC, CNVnator, and IFTV, respectively. Each circus diagram is composed of two parts, the upper part consists of four arcs corresponding to the four detection methods and the lower part consists of 22 arcs corresponding to the 22 autosomes. In the lung cancer diagram, dpCNV obtains the largest number of CNVs while CNVnator obtains the smallest number of CNVs. In the diagrams of the two ovarian cancer samples, CNVnator gets the largest number of CNVs while FREEC and dpCNV get relatively fewer CNVs.

In addition, based on the COSMIC (catalog of somatic mutations in cancer) database, we analyze the CNVs detected by our proposed method on three whole genome sequencing data from biological meanings. For example, 425 CNVs detected by dpCNV from the lung cancer sample are compared to the COSMIC database. There are 151 cytobands and 405 genes in the comparative result. We may notice that many cytobands contain a lot of meaningful genes. For example, the cytoband 11p15.5 contains IFITM1 (Sakamoto et al., 2020) and IFITM3 (Infusini et al., 2015). Many of genes are confirmed to be tumor driver genes and closely related to non-small cell lung cancer, such as C3orf21 (Yang et al., 2017), ZNF454 (Zhu et al., 2020), and C10orf137 (Zheng et al., 2013). For the two ovarian cancer samples, dpCNV gets 225 cytobands and 128 cytobands, 285 genes and 529 genes overlapped with the COSMIC database, respectively, in which there are many important tumor driver genes corresponding to ovarian cancer, such as PUM1 (Guan et al., 2018), GOLPH3L (Feng et al., 2015), PIWIL4 (Guo et al., 2009), and KNDC1 (Yu et al., 2020).

## Conclusion

Accurate detection of CNVs is a crucial step for a comprehensive analysis of genomic mutations in the study of genome evaluation and human complex diseases. In this article, a new method named dpCNV is proposed for the detection of CNVs from NGS data. The central point of dpCNV is that it extracts two meaningful features based on the density peak algorithm and establishes a two-dimensional null distribution to test the significance of genome segments. dpCNV is different from traditional methods and have some new characteristics: (1) it considers the intrinsic correlations among genome bins, and adopts Fused-Lasso segmentation algorithm to smooth the noise data between adjacent bins; (2) it carefully takes into account that CNV regions usually accounts for a small fraction of the whole genome and many CNVs just display a “local” outlier state, and thus extracts two related features (i.e., local density and minimum distance) from the RD profile based on the density peak algorithm; (3) it analyzes the two feature values for each segment through multivariate Gaussian distribution and calculates the corresponding *p*-value to declare whether it is a CNV.

The performance of dpCNV is assessed and validated through simulation studies and applications to a set of real sequencing samples. In simulation experiments, dpCNV outperforms four peer methods (FREEC, GROM-RD, CNVnator, and CNV_IFTV) in terms of sensitivity and F1-score. In real sample experiments, dpCNV is performed on three whole genome sequencing samples including a lung cancer sample and two ovarian samples, and is compared with three peer methods (FREEC, CNVnator, and CNV_IFTV). Here, we have not make comparison with GROM-RD since it has not obtained results from these real sequencing samples. In this comparison, we make an evaluation of the four methods by using ODS. The result indicates that dpCNV obtains a better performance than other methods. In addition, we demonstrate the biological meanings of the detected CNVs by referring the COSMIC database.

With regard to the future work, we plan to make a further improvement to the current version of the dpCNV method from the following aspects. In the first place, we will design a strategy to predict tumor purity and integrate it to the detection of CNVs. In the second place, we intend to predict absolute copy numbers for each CNV region, since absolute copy numbers might provide much information of the study of chromosome instability. In the third place, we intend to combine the detection of CNVs with other types of genomic mutations into a pipeline analysis, which will help to improve the efficiency of genomic mutation analysis. In the last palace, it is necessary to explore the detection of CNVs by using mRNA sequencing data. Generally, RD values obtained from the sequencing data on DNA are closely related with copy numbers. A high expression of mRNAs might be associated with a large copy number. Therefore, using mRNA sequencing data may facilitate the detection of CNVs in tumor genomes.

## Data Availability Statement

The original contributions presented in the study are included in the article/supplementary material, further inquiries can be directed to the corresponding author/s.

## Author Contributions

KX and YT participated in the study and design of algorithms and experiments, and participated in writing the manuscript. XY directed the whole work, conceived of the study and help, and edited the manuscript. YT participated in the analysis of the performance of the proposed method. All authors read the final manuscript and agreed the submission.

## Conflict of Interest

The authors declare that the research was conducted in the absence of any commercial or financial relationships that could be construed as a potential conflict of interest.

## Footnotes

1 https://www.ebi.ac.uk/ega/
